# Thermal burn of palate caused by microwave heated cheese-pie: A case report

**DOI:** 10.1186/1757-1626-1-191

**Published:** 2008-09-30

**Authors:** Panagiotis Kafas, Christos Stavrianos

**Affiliations:** 1 Department of Oral Surgery and Radiology, School of Dentistry, Aristotle University, Thessalonica, Greece; 2 Department of Endodontics, School of Dentistry, Aristotle University, Thessalonica, Greece

## Abstract

A female patient, 36-years-old, complained of bilateral palatal pain on the anatomical area of upper second molars. The painful condition of palatal mucosa erosion was observed. Palatal erosions or ulcerations may be caused by heated food cooked in microwave ovens. We present a case of a bilateral palatal burn caused by cheese-pie. Concluding, any food containing cheese, when heated in microwave oven, may cause palatal burn if eaten immediately.

## Background

Thermal burn of tissues in oral cavity is not commonly seen in dental practice. Adopting the Western culture and life in such matters as technology may be the primary reason for this type of injury. The modern life should include technologies of saving time. Microwave ovens are probably considered the most innovated cooking machines. The cooking properties are different. The risk of human injury is evident [1-3]. There is no doubt that any such equipment should also have negative biological effects. Currently, there are few reports in the English literature emphasizing the possible thermal effect of microwaved eggs, pizzas and liquids on tissues [1,3,4].

## Case Report

**Figure 1:**
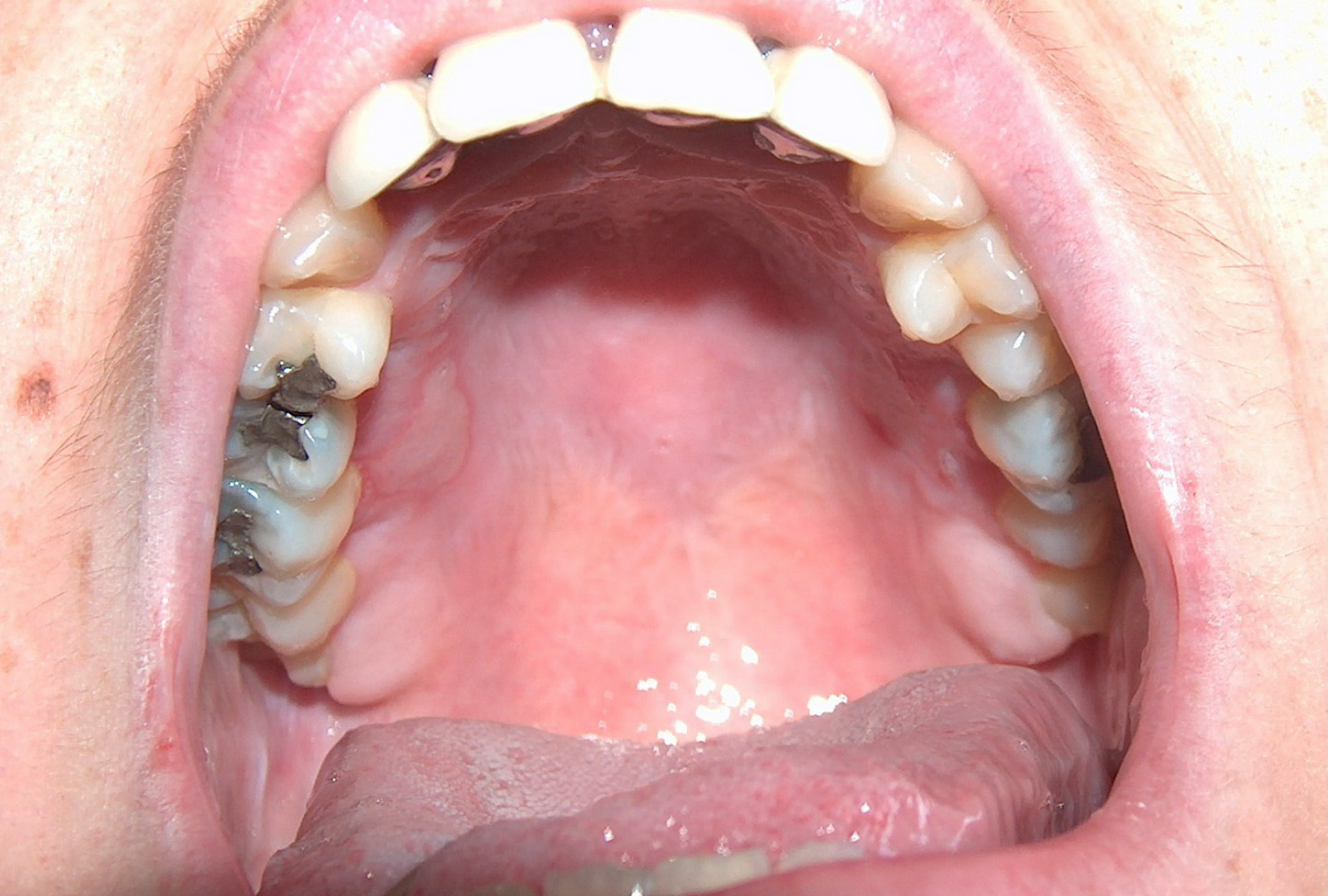
Bilateral palatal burn caused by cheese-pie cooked in microwave oven.

**Figure 2:**
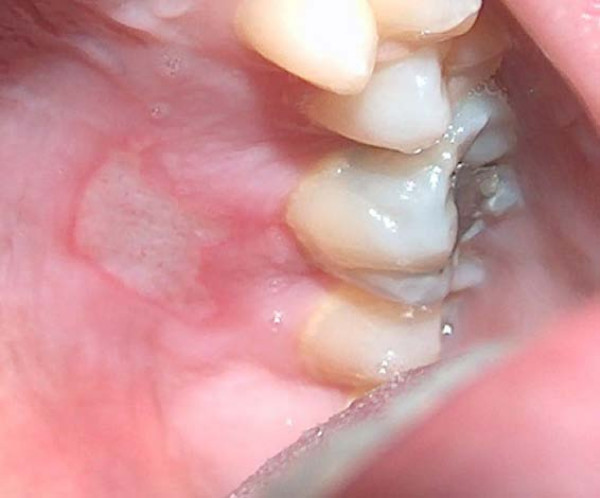
Erosion of the left palatal mucosa.

**Figure 3:**
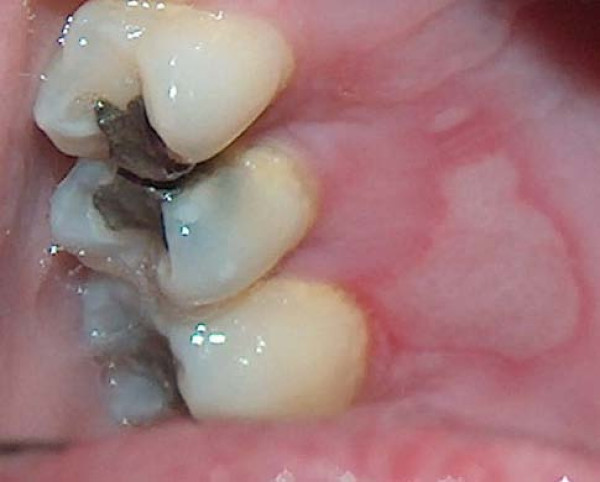
Erosion of the right palatal mucosa.

A Caucasian female patient, 36-years-old presented to the clinic with painful bilateral palate (Figure. 1). The patient works in a financial company. She has been smoking 10 cigarettes per day for 11 years. She only drinks alcohol occasionally. The medical history was free. The dental history was free apart from routine dental procedures, such as fillings and prosthetics. The patient was 64 kg weight and 164 cm height. The burn incident, as described by the patient, happened the moment she bit and chewed a near the center of the pie part, which caused her extreme pain before she could spit the bite out. The still remaining cheese elements in the mouth cavity prolonged the feeling of pain, as they remained extremely hot. The clinical examination that followed revealed erosive lesions of the lateral palate. The lesions could be described as oval-shaped or circular pattern erosions, with erythematous borders surrounding whitish damaged mucosa (Figure. 2, 3).

The recommended treatment included 0.12% chlorhexidine mouth-rinse for one week and preventive measurements. It was suggested to avoid smoking and drinking or eating very hot and acidic foods (vinegar, lemon juice, e.t.c) and alcohol. On the other hand, it was recommended to drink non-carbonized cold fluids and to brush carefully the teeth without injuring the erosive areas.

## Discussion

It is well-known that microwave heated food may cause human injury [1]. Most commonly involved area is considered the palatal arc and the anterior tongue [2]. Usually, pizzas heated in microwave oven cause such palatal burns [3].

The temperature of food heated by microwaves is another important issue for understanding the aetiology of this type of injury. It is generally accepted that food centrally containing liquid or soft material, such as cheese, may have higher temperature internally than externally [2]. This is a well-known cooking property of microwave ovens.

Currently there are few reports of mentioning the danger of eating pizzas, eggs and liquids processed in microwave ovens [1,3,4]. Cheese pies and pizzas have the same main content. Hypothetically, cheese is the material that retains temperature in high levels. In our opinion, cheese pies become more dangerous than pizzas when heated in microwave, because the implicated material is located centrally. In pizzas, the melted cheese may be easily seen indicating that the temperature is still high. In cheese pies, the effect of peripheral cooling does not indicate the same for the central portion. This is dangerous because tactile sensation is not adequate to assess the entire temperature. Biting microwave-heated food allows the entire melted cheese to flow on the mucosa causing areas of erosion, or in more extreme cases, ulcerations. The previous observation depends primarily on the temperature at the time of contact, and secondly the duration of contact.

Management of palatal burns depends on the size of the lesion. Up to medium size lesion, it is recommended to use preventive measurements, such as non steroidal anti-inflammatory drugs (NSAID's), antibiotics – in cases of poor oral hygiene or in cases where systemic status indicates – and finally antiseptic mouth washes. There is no reported case of extensive ulceration of the palate due to heated food in microwave causing mucosa burns.

As a final point, microwaves possibly have the ability to increase kinetic energy of food contents inconsistently. This hypothesis derived from the definition of Boltzmann about temperature: "Is a measure of the kinetic energy of a moving object" [5].

## Conclusion

Individuals using microwave ovens for cooking or heating cheese-pies should be aware that the internal pie temperature may not be the same as the external, which increase the risk of oral mucosa burns. A research study should be carried out to evaluate the temperature of different types of cheese in relation to time and energy used for cooking in microwave ovens.

## Competing interests

The authors declare that they have no competing interests.

## Authors' contributions

CS analyzed the patient data. PK was major contributor in assessing the case and writing the manuscript. All authors read and approved the final manuscript.

## Consent

Written informed consent was obtained from the patient for publication of this case report and accompanying images. A copy of the written consent is available for review by the Editor-in-Chief of this journal.
